# Five-year microevolution of a multidrug-resistant *Mycobacterium tuberculosis* strain within a patient with inadequate compliance to treatment

**DOI:** 10.1186/s12879-021-06069-9

**Published:** 2021-04-29

**Authors:** Darío A. Fernandez Do Porto, Johana Monteserin, Josefina Campos, Ezequiel J. Sosa, Mario Matteo, Federico Serral, Noemí Yokobori, Andrés Fernández Benevento, Tomás Poklepovich, Agustín Pardo, Ingrid Wainmayer, Norberto Simboli, Florencia Castello, Roxana Paul, Marcelo Martí, Beatriz López, Adrián Turjanski, Viviana Ritacco

**Affiliations:** 1 Instituto de Cálculo, Facultad de Ciencias Exactas y Naturales, Universidad de Buenos Aires, Buenos Aires, Argentina; 2 Departamento de Química Biológica, Facultad de Ciencias Exactas y Naturales, Universidad de Buenos Aires, Buenos Aires, Argentina; 3 Instituto Nacional de Enfermedades Infecciosas-ANLIS Carlos Malbrán, Buenos Aires, Argentina; 4 Consejo Nacional de Investigaciones Científicas y Técnicas (CONICET), Buenos Aires, Argentina; 5 Instituto de Química Biológica de la Facultad de Ciencias Exactas y Naturales, IQUIBICEN, CONICET, Buenos Aires, Argentina; 6 Instituto de Tisioneumonología Raúl F. Vaccarezza, Hospital de Infecciosas Dr. F. J. Muñiz, Buenos Aires, Argentina

**Keywords:** Drug resistant tuberculosis, Multidrug-resistance, Clonal evolution, High-throughput nucleotide sequencing

## Abstract

**Background:**

Whole-genome sequencing has shown that the *Mycobacterium tuberculosis* infection process can be more heterogeneous than previously thought. Compartmentalized infections, exogenous reinfections, and microevolution are manifestations of this clonal complexity. The analysis of the mechanisms causing the microevolution —the genetic variability of *M. tuberculosis* at short time scales— of a parental strain into clonal variants with a patient is a relevant issue that has not been yet completely addressed. To our knowledge, a whole genome sequence microevolution analysis in a single patient with inadequate adherence to treatment has not been previously reported.

**Case presentation:**

In this work, we applied whole genome sequencing analysis for a more in-depth analysis of the microevolution of a parental *Mycobacterium tuberculosis* strain into clonal variants within a patient with poor treatment compliance in Argentina. We analyzed the whole-genome sequence of 8 consecutive *Mycobacterium tuberculosis* isolates obtained from a patient within 57-months of intermittent therapy**.** Nineteen mutations (9 short-term, 10 fixed variants) emerged, most of them associated with drug resistance. The first isolate was already resistant to isoniazid, rifampicin, and streptomycin, thereafter the strain developed resistance to fluoroquinolones and pyrazinamide. Surprisingly, isolates remained susceptible to the pro-drug ethionamide after acquiring a frameshift mutation in ethA, a gene required for its activation. We also found a novel variant, (T-54G), in the 5′ untranslated region of whiB7 (T-54G), a region allegedly related to kanamycin resistance. Notably, discrepancies between canonical and phage-based susceptibility testing to kanamycin were previously found for the isolate harboring this mutation. In our patient, microevolution was mainly driven by drug selective pressure. Rare short-term mutations fixed together with resistance-conferring mutations during therapy.

**Conclusions:**

This report highlights the relevance of whole-genome sequencing analysis in the clinic for characterization of pre-XDR and MDR resistance profile, particularly in patients with incomplete and/or intermittent treatment.

**Supplementary Information:**

The online version contains supplementary material available at 10.1186/s12879-021-06069-9.

## Background

Tuberculosis (TB) ranks among the top 10 infectious killers in the world and multidrug-resistant TB (MDR-TB) − TB caused by bacilli resistant to the key first-line anti-TB drugs, isoniazid (INH) and rifampicin (RIF) – is largely responsible for TB mortality [1]. *Mycobacterium tuberculosis,* the causative agent, is an obligate human pathogen that has coevolved with its host and lacks inter-genomic mobility. Thus, unlike other bacterial species, it does not acquire drug resistance by horizontal gene transfer. Instead, it undergoes a stepwise process triggered by the spontaneous emergence of drug-resistant clones followed by fixation of resistant mutants in the bacillary population; transmission of the resistant clones perpetuates them in the community [2]. The primary mechanism underlying *M. tuberculosis* drug resistance is the acquisition of mutations in drug-targets or genes coding drug activating enzymes [3]. Recent work showed that the frequency of mutations causing drug resistance is boosted during drug exposure of the bacilli [4].

The treatment success of MDR-TB relies on case detection, drug susceptibility testing (DST) availability, access to second-line drugs, and prompt initiation of adequate therapy. Traditionally, a history of prior treatment for TB has been deemed as the most common risk factor for MDR-TB. However, growing genotyping evidence indicates that the worldwide MDR-TB epidemic is mainly driven by direct transmission of MDR *M. tuberculosis* strains [5]. Besides, under-recognized and under-treated MDR-TB patients maintain drug-resistant strains in the community for prolonged periods through uninterrupted chains of transmission, thus increasing the risk of spontaneous emergence of additional drug-resistance conferring mutations [6].

Genotyping methods − such as mycobacterial interspersed repetitive units-variable number tandem repeat (MIRU-VNTR) and spoligotyping *−* have disclosed the existence of a wide intra-species genomic diversity in *M. tuberculosis*. These methodologies made it possible to distinguish relapses from reinfections and mixed infections concurring within a single host. More recently, the introduction of whole-genome sequencing (WGS) has changed the way we think about *M. tuberculosis* population dynamics and epidemiology. Nowadays, it is possible to get a deep insight into the timeline of drug resistance acquisition [7, 8], decipher how the transmission events occur, and integrate all the data into a phylogeographic setting [9]. Furthermore, WGS shows that the *M. tuberculosis* infection process can be more heterogeneous than previously thought. Compartmentalized infections, exogenous reinfections, and microevolution processes (i.e. genetic variability in short-time scale) are different versions of this clonal complexity [7, 10]. In particular, the microevolution of a parental *M. tuberculosis* strain into clonal variants within a single patient is a relevant issue to be addressed.

Argentina is a South American country with a moderate burden of TB and disparate rates across the community. Vulnerable groups, such as migrants from neighboring countries, prisoners and previously-treated active cases of MDR-TB and XDR-TB (extensively drug-resistant tuberculosis, i.e. MDR-TB with additional resistance to at least one fluoroquinolone and 1 sec-line injectable drug) are still posing a significant challenge [11, 12]. This case study explores the genomic diversity of *M. tuberculosis* in an Argentinean patient with a history of TB and inadequate adherence to treatment. To understand the evolution of the TB infection in this patient, we performed WGS of 8-time serial isolates obtained during a 57-month period. We report results on the phylogenetic analyses, microevolution, and relation between genomic variants and phenotypic resistance.

## Case presentation

### Patient clinical records

**Fig. 1:**
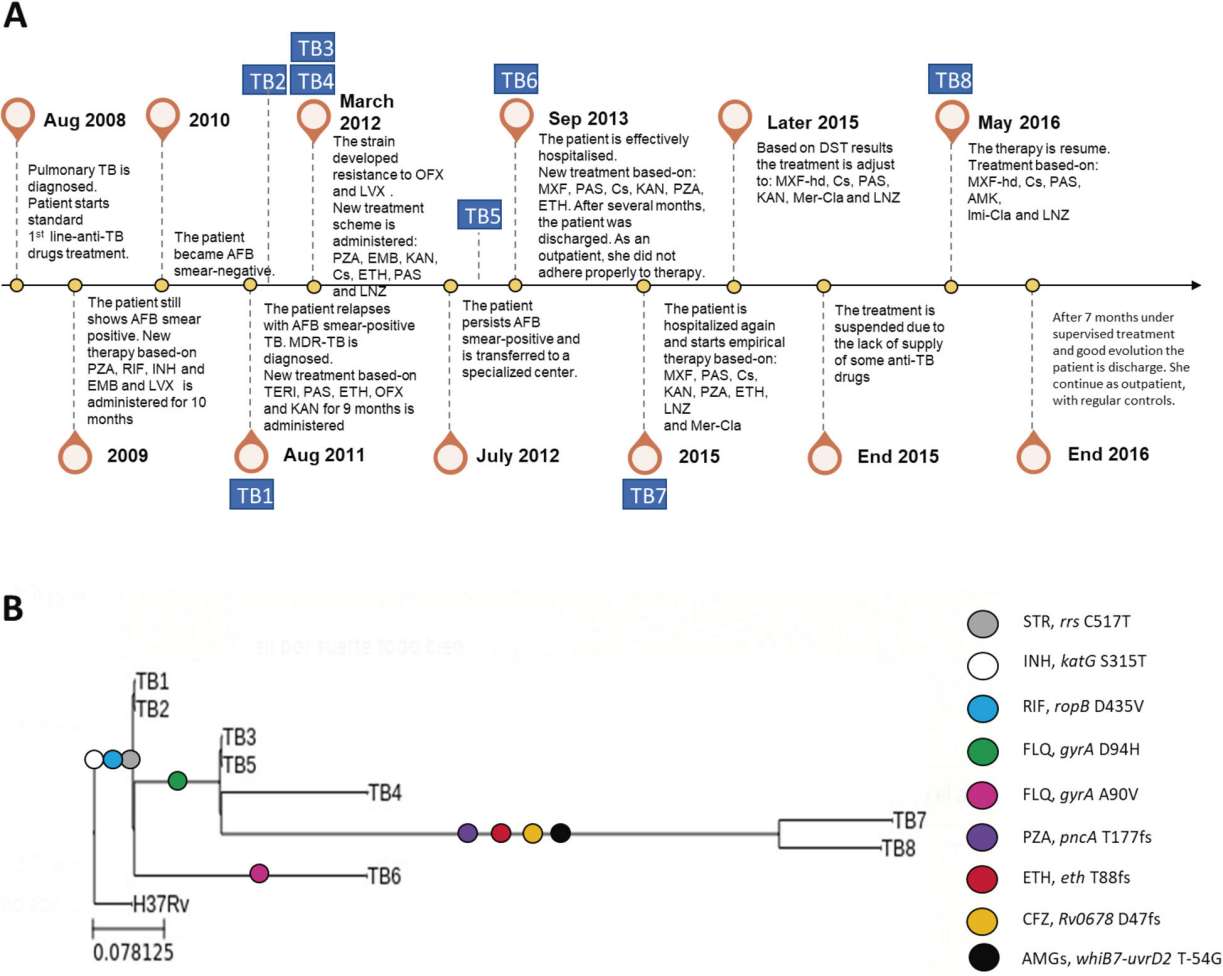
Patient history and clinical isolates. **a**, Patient history and clinical records. **b**, Phylogeny of 8 time-serial isolates with drug-resistance. Resistance-conferring mutations are directly indicated on the tree. TERI: terizidone, PAS: para-aminosalicylic acid, OFX: ofloxacin, KAN: kanamycin, PZA: pyrazinamide, Cs: cycloserine, LNZ: linezolid, MXF: moxifloxacin, MXF-hd: moxifloxacin high dose, IC: imipenem-clavulanate, MC: meropenem-clavulanate AMK: amikacin. STR: streptomycin, INH: isoniazid, RIF: rifampicin, ETH: ethionamide, CFZ: clofazimine, AMGs: aminoglycosides

A 40-year-old HIV-negative woman – who was born in the northwestern region of Argentina and worked as a cleaner in a private health center in Buenos Aires − was diagnosed with pulmonary TB in 2008, based on clinical and radiological data (multiple cavitary lesions) together with the presence of acid-fast bacilli (AFB) in sputum smear examination at Argerich Hospital. The patient started standard anti-TB treatment consisting of 2 months RIF, INH, pyrazinamide (PZA), and ethambutol (EMB), followed by 4 months RIF and INH (Fig. 1a). No data about the tuberculosis status could be obtained at the end of this treatment. In 2009, she returned to Argerich Hospital with AFB-positive sputum and lung cavitation. The treatment started again with RIF, INH, PZA, and EMB plus levofloxacin (LVX) for 10 months. In 2010, the patient became AFB smear-negative and remained so for about a year, denoting that the patient was cured. In August 2011, she relapsed with AFB smear-positive TB. At this point, the first isolate was obtained (TB1) at Muñiz Hospital, showing *M. tuberculosis* resistant to INH, RIF and STR, susceptible to kanamycin (KAN), amikacin (AMK), ofloxacin (OFX), and EMB. LVX minimal inhibitory concentration (MIC) was < 0.5 mg/L. A new drug combination scheme was started, consisting of terizidone, para-aminosalicylic acid (PAS), ethionamide (ETH), OFX, and KAN for 9 months. Three months later, the patient remained AFB smear-positive and culture-positive (TB2), with a DST profile identical to the first one. In March 2012 (TB3, TB4), the patient was still bacteriologically positive, and the isolate showed additional resistance to OFX and LVX (MIC 12 μg/ml). The isolate remained susceptible to PZA, EMB, KAN, AMK, capreomycin (CP), ETH, cycloserine (Cs), PAS, and moxifloxacin (MFX). Based on these results, a new therapy was established with PZA, EMB, KAN, ETH, Cs, PAS, and linezolid (LNZ). In July 2012, the patient persisted AFB smear-positive and was hospitalized in Muñiz Hospital. In December (TB5), the isolate was resistant to INH, RIF, STR, OFX, and LVX and persisted susceptible to KAN, AMK, CP, PZA, PAS, Cs, ETH, LNZ, and MFX.

In September 2013 (TB6), the patient was hospitalized again and was prescribed a second-line treatment scheme with MXF, PAS, Cs, KAN, PZA, and ETH. The ETH was taken irregularly due to intolerance until it was suspended. After 7 months, the patient was discharged from hospital. As an outpatient, she did not adhere properly to therapy.

In 2015 (TB7), the patient returned symptomatic and was readmitted to Muñiz Hospital where she was treated empirically with MXF, PAS, Cs, KAN, PZA, ETH, LZN, and meropenem-clavulanate while waiting for results of a new DST. Once again, the ETH had to be suspended due to intolerance. The DST results showed resistance to INH, RIF, STR, OFX, LVX, PZA and MXF, this latter at 0.5 μg/ml. In view of these results, the drug scheme was adjusted to high-dose MXF, Cs, PAS, KAN, meropenem-clavulanate, and LNZ. After 10 months, this treatment was discontinued due to a supply shortage in the hospital pharmacy. In May 2016 (TB8), the therapy was resumed, but meropenem-clavulanate was replaced by imipenem-clavulanate and AMK was administered instead of KAN. This drug scheme was accomplished under supervision for 7 months. After 152 days of AMK and 209 days of imipenem-clavulanate with favourable evolution, the patient was discharged and injectable drugs were discontinued. She continued treatment as an outpatient under regular controls. By the end of 2016, the patient remained asymptomatic and negative to AFB smear and culture, denoting the patient was completely cured (Fig. 1a).

#### Clinical isolates

We analyzed the 8 available isolates obtained from the same patient between August 2011 and January 2016 at Hospital Muñiz. Identification and DST to first- and second-line anti-TB drugs was performed at Muñiz Hospital and confirmed at the TB National Reference Laboratory (NRL) at the National Institute of Infectious Diseases Dr. Carlos Malbrán (ANLIS). The first 4 primary sputum cultures (TB1 to TB4) were collected at Argerich Hospital, the rest (TB5 to TB8) were collected at Hospital Muñiz; both hospitals are located in Buenos Aires. A previous TB episode occurred in 2008, and by 2010 the patient had become smear-negative and was discharged. Unfortunately, no isolate was available from that first episode, and we cannot determine if the following TB episode in August 2011 was caused by the same strain.

#### Microbiological and molecular studies

All isolates were grown on Löwenstein-Jensen slants and identified as *M. tuberculosis* by biochemical and molecular tests. DST was performed by the reference standard proportion method in Löwenstein-Jensen medium and/or BACTEC MGIT 960 (Becton Dickinson, MD) under international standards [13]. A multiplex allele-specific PCR (MAS-PCR) for detection of mutations conferring resistance to INH and RIF (codons *katG*315, *inhA*-15, rpoB450, 445, and 425) was performed on all isolates according to a modified protocol described elsewhere [14].

Genotyping was performed by spoligotyping and MIRU-VNTR according to standard procedures [15, 16], followed by comparison with SITVITWEB [17] and MIRU-VNTRplus database [18].

#### Genome sequencing

To perform WGS, we re-cultured the isolates on Löwenstein-Jensen slants. The DNA was extracted according to a standard protocol for mycobacteria [19]. Genomic libraries were constructed using Nextera® XT DNA Sample Preparation Kit (Illumina) following the manufacturer’s instructions. Individual libraries were indexed with Nextera® XT Index Kit. Paired-end reads from all the isolates were obtained using the Illumina MiSeq platform at ANLIS. All reads were deposited in SRA NCBI, Accession number PRJNA646920.

#### Variant calling

Raw reads were checked for quality using FastQC version v0.11.5 [20] and processed with the PRINSEQ lite version 0.20.4 (reads shorter than 36 bp and mean Q < 20 were filtered out) [20, 21] ensuring quality assessment. The reads were aligned to the H37Rv reference genome (NCBI access number NC_000962.3) using BWA 0.7.17 [22]. Variant calling was made with GATK 4.1.8.0 [23]. After filtering problematic regions, we applied 3 different approaches to the analysis: first, we called variants at a low frequency (0.25) to explore short-term mutations, then we called variants at a higher frequency (0.75) to infer phylogenetic trees. Finally, we called even more frequent variants (0.9) to identify fixed mutations within bacillary populations. The resulting variants were annotated with SnpEff 4_3t [24] and compared with our database of drug resistance-associated variants which combines data of TB Profiler v2.8.8 [25], KvarQ v0.12.2 [26], CARD RGI 5.1.1 (database downloaded on June 18, 2020) [27], and bibliographic data. The analysis of the variant-containing genes was performed with Target Pathogen [28].

#### Phylogenetic analyses

The phylogenetic analysis was performed by comparing the samples TB1 to TB8 with representative samples of the main *M. tuberculosis* lineages and sublineages [29] downloaded from NCBI. The reads were processed using the variant calling pipeline mentioned above. The resulting variants from all samples were combined in a single VCF using GATK. To confirm that all the patient’s isolates composed a single monophyletic clade, we constructed a maximum-likelihood genome phylogenetic tree using RaXML v8.2.12 [30] dataset used includes 1 or more representative genomes for each *M. tuberculosis* sublineage.

Nineteen mutations emerged among the 8 time-serial isolates over 57-month of intermittent therapy. Nine out of these 19 variants reached fixation in the bacilli population. The other 10 were categorized as short-term mutations, i.e. single nucleotide polymorphism (SNPs) and Insertion–deletion mutations (InDels) that arise and fade-out within months or years (Supplementary Table 1). The most recent common ancestor was MDR and evolved to a pre-XDR status within 7 months (Fig. 1b).

**Fig. 2:**
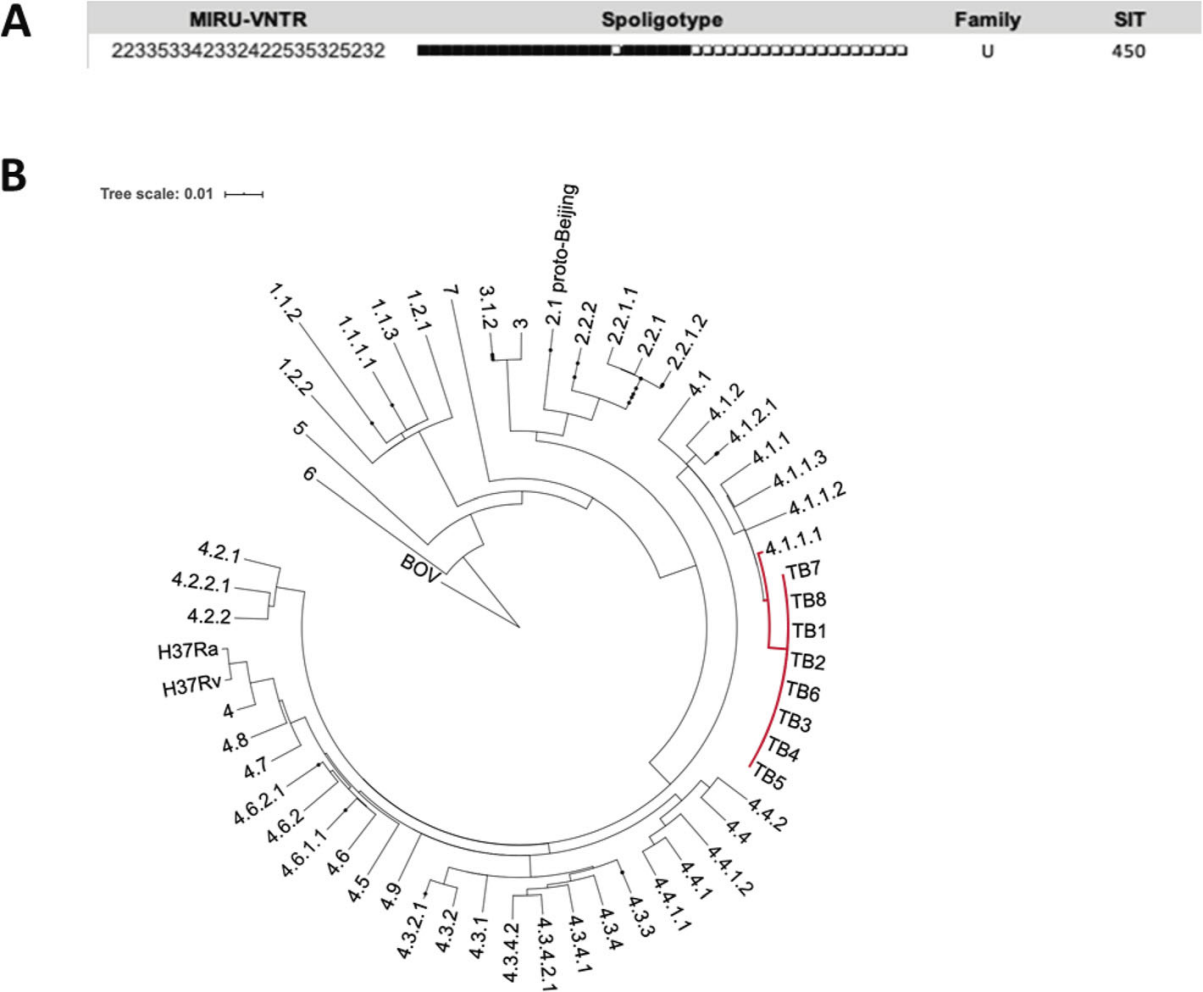
**a**. MIRUs-VNTR and spoligotype patterns displayed by the eight time-serial isolates. **b**. Phylogenetic reconstruction based on the eight time-serial isolates analyzed and 193 genomes representative of all *M. tuberculosis* lineages and sub-lineages according to SNP barcode classification by Coll et al. [31]

The clonal nature of the isolates was supported by identical MIRUs-VNTR and spoligotyping patterns (Fig. 2a). Spoligotyping results showed that all the samples belonged to SIT450, an ambiguous clade between X1 and T5. These sublineages belong to the 4 Euro-American lineage, which is the predominant in Latin America [32].

To place the patient’s isolates in a global phylogenetic context, and confirm they form a single monophyletic clade, we constructed a maximum-likelihood core genome phylogenetic tree. According to a recent SNP-based phylogenetic classification [31], the 8 isolates composed a monophyletic cluster assigned to the sub-lineage 4.1.1, which corresponds to the X type (Fig. 2b).

#### Drug-resistance conferring mutations and putative compensatory mutations

In August 2011, the TB NRL received the first isolate (TB1), and 3 months later the second one (TB2). Both isolates were found to be already resistant to INH and RIF – that is, they qualified as MDR – with additional resistance to STR. These isolates harbored *katG* S315T, *rpoB* D435V, and *rrs* C517T, mutations related to resistance to these three drugs, respectively. These 3 mutations remained fixed in all 8 isolates until the end of the treatment. The genotypic prediction of resistance from WGS data was in line with the results of the phenotypic and molecular techniques applied at the TB NRL (Table 1 and Supplementary Table 2).

**Table 1:** Drug resistance conferring-mutations and their correlation with phenotypic drug resistance

Antibiotic	Gene	Amino acid change	Phenotypicresistence	Genome coverage	Mutation frequency	Isolate
INH	*katG*	S315T	yes	95x	1	TB1–8
RIF	*rpoB*	D435V	yes	102x	1	TB1–8
STR	*Rrs*	C517T	yes	147x	1	TB1–8
OFX- LVX- MXF	*gyrA*	D94H	yes	71x	1	TB3–5, TB7–8
OFX- LVX	*gyrA*	A90V	yes	77x	0.85	TB6
PZA	*pncA*	T177fs	yes	47x	1	TB7–8
ETH	*ethA*	T88fs	no	53x	0.6	TB7–8
AMGs	*5’utr-whiB7*	3568733A > C	no	73x	1	TB7–8
CFZ- BDQ	*Rv0678*	D47fs	yes (CFZ)	45x	0.5	TB7–8

*INH* Isoniazid, *RIF* Rifampicin, *STR* Streptomycin, *OFX* Ofloxacin, *LVX* Levoflaxin, *MXF* Moxifloxacin, *PZA* Pyrazinamide, *ETH* Ethionamide, *AMGs* Aminoglycosid, *CFZ* Clofazimine, *BDQ* Bedaquiline

**Fig. 3:**
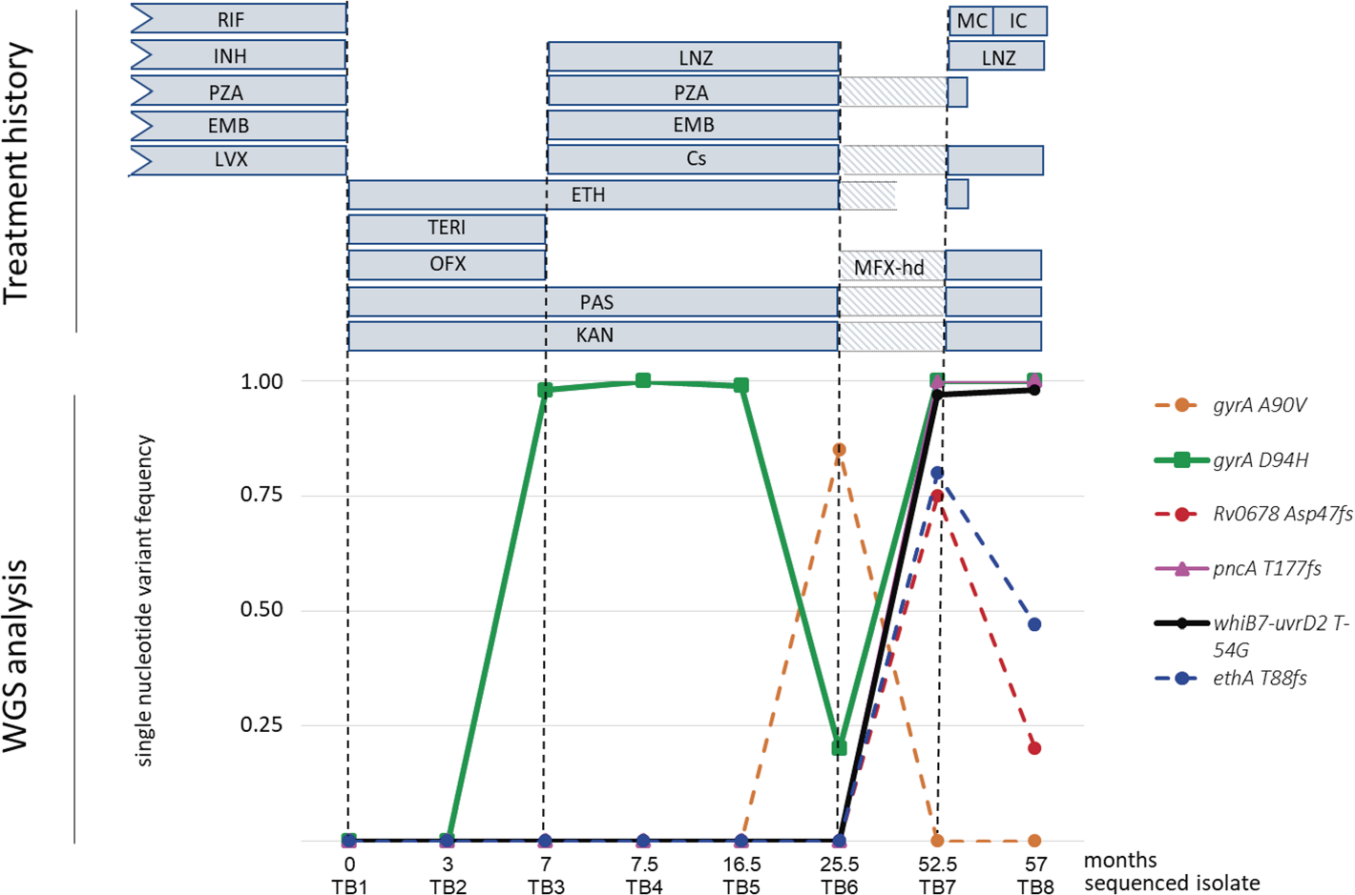
Frequency of drug-resistance conferring mutations along with treatment history. TERI: terizidone, PAS: para-aminosalicylic acid, OFX: ofloxacin, KAN: kanamycin, PZA: pyrazinamide, Cs: cycloserine, LNZ: linezolid, MXF: moxifloxacin, MXF-hd: moxifloxacin high dose, IC: imipenem-clavulanate, MC: meropenem-clavulanate AMK: amikacin. Continue-line: fixed mutations, dashed lines: non fixed mutations

By March 2012 (TB3), phenotypic resistance to OFX and LVX was detected, with borderline resistance to MXF, which changed the strain status to pre-XDR. This phenotypic resistance was supported by the mutation *gyrA* D94H. The strain remained phenotypically resistant to fluoroquinolones until the end, but the frequency of this mutation fluctuated between isolates. In month 25 of treatment (TB6), we registered a decrease in the frequency of clones with *gyrA* D94H mutation and the rise of a new population of clones carrying *gyrA* A90V mutation (Fig. 3). Taking advantage of the closeness of the 2 variant positions, we examined whether the same clonal variant harboured these 2 SNP simultaneously. We did not find evidence of a single clonal variant harbouring these 2 variants together in any of the 97 reads that shared both positions. Clones carrying *gyrA* A90V vanished in subsequent isolates along with the implementation of adequate therapy. Although we detected both mutations 15 months apart, these resistance conferring-mutations emerged independently from the parental MDR strain (Fig. 1b and Fig. 3).

When the therapy was resumed (52 months of treatment, TB7), we observed an increased genetic heterogeneity, involving both, short-term and fixed drug-resistance conferring mutations. Among the short-term SNPs, we found 2 mutations related to drug resistance, *ethA* (T88fs) and *Rv0678* (N47fs). Mutations in the *ethA* gene have been reported to confer resistance to ETH [33]. More than half of the reads of TB7 and TB8 harboured an ethA frameshift mutation but these last 2 isolates remained notably susceptible to ETH. The patient’s drug scheme included ETH, but she took it irregularly due to intolerance until it was suspended. The *Rv0678* gene – where we found a frameshift – is a transcriptional repressor of the genes encoding the MmpS5-MmpL5 efflux pump, which has been associated with bedaquiline (BDQ) and clofazimine (CFZ) resistance.

Among the drug resistance-conferring mutations that became fixed, one appeared towards the end of the treatment at the *pncA* gene (T177fs), confirming the phenotypic resistance to PZA. Another was *gyrA* D94H, which finally re-emerged and became fixed in the bacillary population (TB8) (Fig. 3). A mutation in the 5′ untranslated region of *whiB7* gene (T-54G) was found in the last 2 isolates (TB7 and TB8). The *whiB7* gene is a transcriptional activator of the *eis* gene. Mutations in its 5′ untranslated region have been related to *whiB7* overexpression and the subsequent increase in *eis* expression, ultimately conferring resistance to KAN [34].

### Within-host diversity/microevolution

To explore the within-host diversity among the 8 time-serial isolates, we called variants at a frequency of 25%. Along with the aforementioned short-term and fixed mutations related to drug resistance phenotypes, we found other 12 genomic variants: 6 changes were miss-sense mutations, 3 were InDels and 3 were synonymous mutations. (Supplementary Table 1). The overall substitution rate (5.68 SNPs per genome per year) was higher than the estimate for *M. tuberculosis* (0.4–0.5 SNPs per genome per year) [35]. This is in line with previous reports suggesting that antibiotics can distort the mutation rate, as random SNPs emerging in the genetic background of resistant clones could potentially fix together with well-established resistance-conferring mutations during the course of the treatment [4].

Looking at the identity of relevant variants, we found miss-sense mutations and InDels related to intermediary metabolism and respiration (*oplA* V674fs, *Rv2141c* T178A), a stress protein (*Rv1636* D15A), lipid metabolism (*pks6* G807C), and information pathways (*recC* A773D) (Supplementary Table 1), reinforcing the idea of within-host coexistence of different bacillary sub-populations of a single strain.

## Discussion and conclusions

We studied 8 time-serial *M. tuberculosis* isolates from a patient with a previous TB history over 57 months of irregular treatment. According to the spoligotyping classification, all isolates belonged to the international shared type (SIT) 450, which has different geographic representation in the Americas, Europe, and Africa (http://www.pasteur-guadeloupe.fr:8081/SITVIT2/servletTools, last acceded 23rd March 2020). In Argentina, SIT 450 is present in low frequency in drug-susceptible and resistant *M. tuberculosis* clinical isolates [36]. Complete conservation of MIRU-VNTR and spoligotyping pattern was observed trough all samples. In line with the well-documented clonal stability of these markers, is the most likely situation among serial isolates from a single infection [37]. Moreover, the patient was born in Argentina, a country out of the list of high burden countries and had no known risk of TB exposure. These features make unexpected a mixed or superinfection scenario, typically seen in high incidence populations or high-risk settings [38]. In order to confirm that all isolates form a single monophyletic clade, we constructed a maximum-likelihood core genome phylogenetic tree. According to a recent SNP barcode-based phylogeny, all 8 isolates belong to L4 − the Euro-American lineage amply predominant in Latin America − and compose a monophyletic cluster within the barcode 4.1.1, which corresponds to the X type [31]. Taken together, these data strongly support intrapatient microevolution in a clonal population from a single infection.

In our patient, the microevolution process was mainly driven by short-term mutations which emerged and vanished along with variations in treatment efficacy and compliance. The most recent common ancestor was already MDR and evolved to pre-XDR over 7 months due to an inadequate treatment scheme.

Fluoroquinolone resistance, backed by the gyrA D94H substitution, was the earliest second line resistance to emerge, probably as a result of therapy including OFX and the consequent selection of resistant clones within the bacillary population. The *gyrA* D94H mutation confers high levels of resistance to LVX and MXF. In turn, the *gyrA* A90V mutation causes full resistance to LVX and low-levels or no resistance to MXF, which is still useful at high doses. In our case, even at the point when a borderline phenotypic resistance to MXF was detected, MXF was not excluded from the therapy scheme. This could be a reason why the clones harboring D94H mutation were selected within the bacillary population over the following months. The presence of 2 different clones harboring different fluoroquinolones resistance conferring-mutations supports the hypothesis of within-host independent microevolution events in the bacillary population. This is expected to occur in long-term therapy such as the case described here, in which the different drugs schemes successively administered, and the irregular treatment adherence shaped the evolution of bacilli subpopulations. This applies particularly to chronic recurrent pulmonary TB, which often involves multifocal lesions with different degrees of evolution and drug penetration. Within a single host, *M. tuberculosis* might benefit from this dynamic spatial and temporal heterogeneity by allowing the emergence of alternative drug-resistant variants according to the selective pressure exerted at different times in the different lesional microenvironments [39].

Allegedly, mutations in Rv0678 confer cross-resistance to BDQ and CFZ [40]. We detected a variant with a frameshift (N47fs) in this gene, but those drugs had never been administered to our patient. BDQ has been barely used in Argentina and was not available to the National TB Program at the time of the study. For this reason, we could not test the isolates for phenotypic susceptibility to this drug. On the other hand, CFZ is being included in M/XDR-TB drug schemes. We performed DST for CFZ and found the isolates carrying the mutation to be phenotypically resistant to CFZ (Supplementary Table 2). Variants in the Rv0678 gene were already described in BDQ and CFZ naïve patients in other settings [41, 42]. An explanation for these findings could be the emergence of random mutations in the within-host bacillary populations that were selected with the antibiotic therapy administered. This view is supported by the work of Villelas et al. [41] who found that Rv0678 variants associated with CFZ and BDQ resistance were more frequent in MDR isolates than in drug susceptible isolates from CFZ and BDQ naïve TB patients.

To our knowledge, the variant T-54G found herein in whiB7, had not been previously described, but a variant in the immediate previous position, G-55A, was reported to confer resistance to KAN [34]. TB8, one of the isolates carrying the novel *whiB7* variant, was the subject of a noticeable discrepancy between results of gold standard DST methodologies and a phage-based technique [43]. This isolate resulted susceptible to KAN when tested by the proportion method and MIC (0.5 mg/L) at the TB NRL but was classified as resistant by a novel fluoro-mycobacteriophage reporter method when performed at Hospital Muñiz. We speculate that mutation T-54G in *whiB7* might be associated with low-level resistance to KAN missed by standard growth-based methods, as described for certain non-canonical *rpoB* mutations in relation to phenotypic RIF resistance [44].

Another interesting variant was G807C in *pks6* found in TB6. The *pks* gene family codifies for diverse polyketide synthases. This gene family was shown to have a wide within-host variation [43]. It was proposed that the unusual variability of these genes could allow *M. tuberculosis* to manipulate the host cell during infection. In turn, it is disease severity what seems to drive within-host diversity of pks genes rather than *M. tuberculosis* lineage, TB treatment, or drug resistance status [45].

It is common knowledge that incomplete, intermittent, or inadequate treatment promotes the development of MDR- and XDR-TB. Notably, some recent reports from high-income countries where TB has low incidence have described microevolution leading to drug resistance even in single patients with treatment adherence [4, 46]. In medium and low-income countries, the situation is more propitious to drug resistance. Even where anti-TB drugs are available, in most developing countries TB treatment programs are deficient, usually because of poor treatment compliance of patients and failure to carefully sustain drug supplies and observed treatment. The genomic scrutiny of isolates from TB patients in this context can contribute to a better understanding of the evolution mechanisms of *M. tuberculosis* strains leading to drug resistance. Based on this work and previous data, we emphasize and recommend adequate treatment as preventing the emergence of MDR and XDR TB.

## Supplementary Information

**Additional file 1: Supplementary Table 1**. Within-host microevolution of eight time-serial isolates from a patient with multidrug-resistant tuberculosis and inadequate compliance to treatment

**Additional file 2.**

## Data Availability

The dataset supporting the conclusions of this article is available in the SRA NCBI repository [Accession number PRJNA646920, https://www.ncbi.nlm.nih.gov/bioproject/PRJNA646920].

## Abbreviations

AFB: Acid-fast bacilli
AMGs: Aminoglycosides.
AMK: Amikacin
ANLIS: National institute of infectious diseases Dr. Carlos Malbrán
CFZ: Clofazimine
Cs: Cycloserine
DST: Drug susceptibility testing
EMB: Ethambutol
ETH: Ethionamide
IC: Imipenem-clavulanate
InDels: Insertion–deletion mutations
INH: Isoniazid
KAN: Kanamycin
LNZ: Linezolid
LVX: Levofloxacin
MC: Meropenem-clavulanate
MDR-TB: Multidrug-resistant tuberculosis
MIRU: Mycobacterial interspersed repetitive units
MXF: Moxifloxacin
NRL: National reference laboratory
OFX: Ofloxacin
PAS: Para-aminosalicylic acid
PZA: Pyrazinamide
RIF: Rifampicin
SIT: International shared type
SNP: Single nucleotide polymorphism
STR: Streptomycin
TB: Tuberculosis
TERI: Terizidone
VNTR: Variable number tandem repeat
XDR-TB: Extensively drug-resistant tuberculosis
